# Low-Frequency rTMS over Contralesional M1 Increases Ipsilesional Cortical Excitability and Motor Function with Decreased Interhemispheric Asymmetry in Subacute Stroke: A Randomized Controlled Study

**DOI:** 10.1155/2022/3815357

**Published:** 2022-01-05

**Authors:** Ka Yan Luk, Hui Xi Ouyang, Marco Yiu Chung Pang

**Affiliations:** ^1^Department of Rehabilitation Sciences, Hong Kong Polytechnic University, Hong Kong, China; ^2^Physiotherapy Department, Queen Elizabeth Hospital, Hong Kong, China

## Abstract

**Objective:**

To determine the long-term effects of low-frequency repetitive transcranial magnetic stimulation (LF-rTMS) over the contralesional M1 preceding motor task practice on the interhemispheric asymmetry of the cortical excitability and the functional recovery in subacute stroke patients with mild to moderate arm paresis.

**Methods:**

Twenty-four subacute stroke patients were randomly allocated to either the experimental or control group. The experimental group underwent rTMS over the contralesional M1 (1 Hz), immediately followed by 30 minutes of motor task practice (10 sessions within 2 weeks). The controls received sham rTMS and the same task practice. Following the 2-week intervention period, the task practice was continued twice weekly for another 10 weeks in both groups. Outcomes were evaluated at baseline (T0), at the end of the 2-week stimulation period (T1), and at 12-week follow-up (T2).

**Results:**

The MEP (paretic hand) and interhemispheric asymmetry, Fugl-Meyer motor assessment, Action Research Arm Test, and box and block test scores improved more in the experimental group than controls at T1 (*p* < 0.05). The beneficial effects were largely maintained at T2.

**Conclusion:**

LF-rTMS over the contralesional M1 preceding motor task practice was effective in enhancing the ipsilesional cortical excitability and upper limb function with reducing interhemispheric asymmetry in subacute stroke patients with mild to moderate arm paresis. *Significance.* Adding LF-rTMS prior to motor task practice may reduce interhemispheric asymmetry of cortical excitabilities and promote upper limb function recovery in subacute stroke with mild to moderate arm paresis.

## 1. Introduction

Restoring upper limb function remains a challenging area in stroke rehabilitation. At three to six months poststroke, more than 50% of patients continue to live with residual impairment in arm function [1].

Repetitive transcranial magnetic stimulation (rTMS) has gained increasing attention for its potential clinical application in stroke rehabilitation due to its ability to modulate cortical excitability [2–10]. According to the interhemispheric imbalance model, motor recovery is limited by the presence of asymmetry in interhemispheric inhibition following stroke, with excessive inhibition from the contralesional on the ipsilesional hemisphere [11]. Some studies found that low-frequency rTMS over the contralesional M1 region could suppress overactivity in the contralesional hemisphere, thereby reducing its inhibition on the ipsilesional hemisphere [12, 13]. The restoration of interhemispheric balance was thought to be a potential mechanism underlying the motor recovery of the paretic upper limb [3, 5, 12, 13].

Increasing research has focused on the “priming” effect of rTMS [13–15]. The increase in excitability of the ipsilesional motor cortex induced by low-frequency rTMS over the contralesional hemisphere is related to the modulation of the GABAergic-mediated interhemispheric inhibition [16]. It creates a more favorable environment that may allow the surviving neurons to reorganize in response to motor task practice, thereby enhancing its efficacy to facilitate use-dependent plasticity that is important for motor relearning during stroke recovery [9, 13, 16, 17]. The after-effects induced by rTMS (1 Hz or 5 Hz, 1500-2500 pulses, 90%rMT) on M1, which may stem from changes in synaptic efficacy related to the phenomenon of long-term potentiation and long-term depression, can persist for up from 60 min to 2 hours [18]. Therefore, the timing of the rTMS in relation to the motor training is important. In chronic stroke patients, Avenanti et al. showed that inhibitory rTMS over the contralesional motor cortex preceding the motor training resulted in stronger improvements in motor function, compared with the same rTMS protocol applied after the motor training [16]. However, of the studies in subacute stroke, four reported that inhibitory rTMS over the contralesional motor cortex combined with motor training resulted in better recovery of motor function compared with motor training alone [13, 14, 19, 20], while the others reported negative findings [15, 21].

Recent evidence suggested that the interhemispheric inhibition model does not apply to all patients with stroke [22]. Dodd et al. suggested that the contralesional hemisphere appears to play a key role in motor recovery for at least a subset of stroke patients and that its role could be influenced by severity of disability, stroke duration, and lesion location [22, 23]. This may partly explain the discrepancies in previous research findings. In addition, only one inhibitory rTMS trial in subacute stroke assessed the association between functional recovery and changes in cortical excitability [13]. More research is required in this area.

Another important issue is related to the long-term effects of inhibitory rTMS. Only three studies had a follow-up period that was greater than 8 weeks to test the long-term treatment effect of inhibitory rTMS among individuals with subacute stroke [24]. Of these, Seniow et al. [21] did not measure cortical excitability at all, whereas Lüdemann-Podubecka et al. did not measure the cortical excitability of the ipsilesional side [25]. Motor training immediately after rTMS was not included in Blesneag et al. to explore its priming effect, nor was motor training incorporated during the follow-up period after the rTMS intervention period had ended [26]. More research is thus required to examine the long-term effect of low-frequency rTMS combined with motor task practice.

The objectives of this study were to investigate the effects of a combination of low-frequency rTMS over the contralesional M1 and repetitive upper limb motor task practice on cortical excitability and motor function in subacute stroke patients with mild to moderate motor impairment, when compared to sham stimulation combined with the same motor task practice. It was hypothesized that (1) the former protocol would be more effective in increasing the excitability of the ipsilesional motor cortex and improving motor function of the affected upper limb when compared with the latter treatment after 2 weeks of intervention in subacute stroke patients with mild to moderate motor impairment and that (2) the improvement in outcomes gained (if any) from the 2-week treatment period would be well maintained after 10 weeks of continued motor task practice without rTMS.

## 2. Methods

### 2.1. Study Design

**Figure 1:**
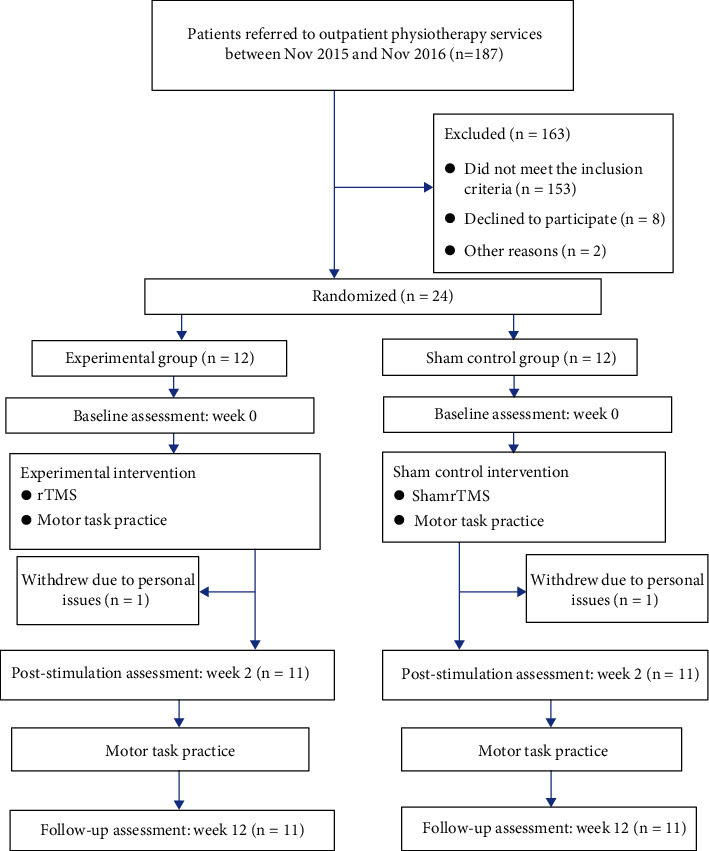
Study flowchart.

This was a double-blind randomized controlled trial (https://clinicaltrials.gov/, registration number: NCT 02490371) [27].  Figure 1 illustrates the overall study design.

### 2.2. Participants

A convenience sampling method was used. Participants were recruited from a local hospital by an independent researcher. The participants were originally admitted to the hospital as inpatients because of stroke and later referred by the physician to receive outpatient physiotherapy service of the same hospital upon discharge. The inclusion criteria were first stroke, subacute stroke (1 month < stroke duration < 6 months), aged 60 or more because stroke was more prevalent in this group than their younger counterparts [28], and muscle strength of the paretic hand and fingers at grade < 5 and >2 according to the Medical Research Council scale [29] because we aimed to recruit stroke patients who had mild to moderate weakness in the affected upper limb, because this subgroup of the stroke population may benefit more from the rTMS intervention [7, 8, 14, 18, 19]. A number of previous research studies on rTMS also targeted those who sustained mild to moderate weakness in their sampling process [2, 14, 19, 21, 30–32]. An MRC grade of 5 is indicative of normal muscle strength. There would be relatively less room for individuals with normal muscle strength to have further improvement in motor function after intervention. Including these individuals may potentially dilute the overall treatment effect of the group. The presence of muscle weakness was also one of the inclusion criteria commonly found in previous rTMS trials in stroke [2, 14, 32]. On the other hand, individuals with a muscle strength grade of ≤2 (i.e., no antigravity movements) would not be able to perform the upper limb tasks used in our protocol due to the severe weakness. Previous work also suggested that rTMS might be less effective for those with severe paralysis [22]. Another inclusion criterion was the ability to carry out normal conversations, because it was important for the participant to be able to understand the rTMS and motor task practice procedures, and inform the researcher of any adverse symptoms during the assessment and training sessions. The exclusion criteria were other neurological conditions except for stroke (e.g., dementia and mental illness), contraindications to rTMS according to guidelines formulated by Wassermann [33], and unstable cardiopulmonary condition. The details of the sample size estimation can be found in Supplementary I.

### 2.3. Randomization

The recruited individuals were randomly assigned to either the experimental group or the control group by drawing a preset-sealed opaque envelope. The randomization sequence was determined by using a table of random numbers, with a block size of 6 and an allocation ratio of 1 : 1. The randomization procedures were performed by an independent researcher. This study followed the principles of the Declaration of Helsinki. Written informed consent was obtained prior to data collection. The trial protocol was approved by the Research Ethics Committee of the involved hospital (approval number: KC/KE-15-0130/FR3) and university (approval number: HSEARS2015318001-01).

### 2.4. Determination of Stimulation Site

All TMS procedures took place in the TMS suite located at a local hospital. The participants were seated in an inclined chair with both the hand and the neck well supported. The hotspots over the primary motor cortex in both hemispheres (M1) were identified in the baseline assessment session, using the Magstim Rapid Stimulator (Magstim Company, Whitland, UK). The stimulator was a figure-of-eight coil (each loop 70 mm in diameter) that was connected to a neuronavigation system (Brian-sight System2; Rogue Research Inc., Montreal, Canada). After proper skin preparation, two electromyography (EMG) electrodes (3M Health Care, St. Paul, USA) were placed over the muscle belly of the first dorsal interosseous (FDI) on each side while the ground electrode was placed over the ulnar styloid process.

The hotspot and motor threshold of the contralesional or ipsilesional M1 were determined by placing the coil tangentially to the scalp over the area of the respective M1. The stimulation site (hotspot) was determined as the location where application of TMS at a slightly suprathreshold intensity induced the highest amplitude of MEP in the FDI. The resting motor threshold (rMT) was defined as the intensity that elicited the MEP at a level of >50 *μ*V in at least 5 of 10 consecutive stimulations. If the EMG could not be triggered at 100% of the stimulator output, the MEP was defined as “cannot be triggered” and set at zero for data analysis purpose. If the MEP of the paretic hand was absent when stimulating the ipsilesional hemisphere, the motor hotspot was defined as being symmetrical to the contralesional hemisphere [13]. To ensure the accuracy of the stimulation site for subsequent treatment sessions, a navigation system was used. Two tracer markers were positioned and secured on the forehead of the participant and on the stimulating coil (Supplementary II). A skull model was recreated from the software and configured with several anatomical landmarks of individual participants, including the bridge of the nose (nasion), the tip of the nose, and the ear (notch above the tragus). The location of the stimulation could then be identified and recorded in the system. The electromyography signals were amplified (2500 V/V), bandpass filtered (1-5 k Hz), and digitized for recording with the ADC sampling rate of 3 kHz by a built-in EMG device in the system.

### 2.5. Experimental Intervention

The experimental group underwent rTMS over the hotspot of the contralesional M1 on weekdays over a 2-week period (i.e., 10 sessions). The protocol used was at a low frequency of 1 Hz with a stimulus intensity of 90% of rMT of the nonparetic hand for a total of 1200 pulses in each session. The same stimulator output was used throughout the 2-week intervention period. This low-frequency protocol was adopted because it was shown to result in downregulation of motor cortical excitability (i.e., inhibitory) over the contralesional cortex [3, 4]. All rTMS treatment was conducted by a physiotherapist who had received formal TMS training and had more than 5 years of experience in neurological rehabilitation.

**Figure 2:**
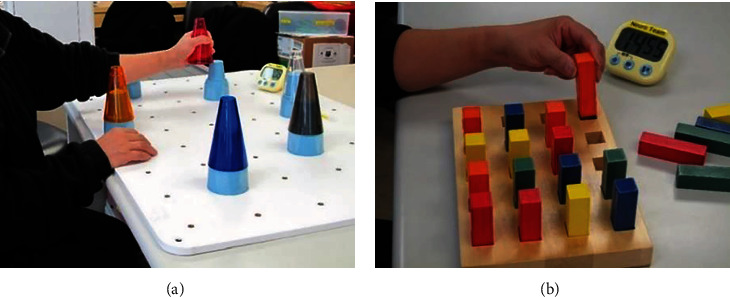
Motor task practice. Two motor tasks were involved: (a) A pegboard with five targets of the same size placed in a semicircle at arm's length was used. Participants were asked to grasp one of the five cones placed in the middle near the edge of the table and then reach and place it on top of the specific target that fitted the size of the cone. The same task was repeated until all five cones were placed on their respective targets. Participants were then required to reach and grasp the cones one by one and place them back to the original position in the middle. These movement sequences were repeated until the 15-minute mark. (b) A pegboard with rectangular blocks was placed in front of the participants, who were then asked to take the block one by one out of the pegboard and then put on the table. After all the blocks were removed from the pegboard, the participants were required to put the block one by one back onto the pegboard. These movement sequences were repeated until the 15-minute mark (Figure 2). In both motor activities, participants were encouraged to perform the tasks as efficiently as possible, without dropping the object on the table.

Immediately after each rTMS treatment session, participants in the experimental group also underwent 30 minutes of motor task practice. The task practice sessions were supervised by a physiotherapy assistant who was blinded to group allocation and included the repetitive practice of two motor tasks (Figure 2).

After the 10 rTMS and motor task practice sessions, the participants continued to undergo the same task practice program twice weekly for 10 weeks.

### 2.6. Control Intervention

The control group received sham rTMS. All procedures were the same as the experimental group, except that sham rTMS was given. Sham stimulation was conducted by positioning the coil at an angle of 90 degrees relative to the scalp instead of tangentially to the hotspot, but the coil produced the same sounds as in real rTMS [13]. This strategy allowed the magnitude of the field delivered to be decreased but did not eliminate it [34] and created a similar sensation. Similar to the experimental group, participants in the control group also underwent 30 minutes of the same motor task practice immediately after each sham rTMS intervention, and also twice weekly for another 10 weeks after the end of the initial 2-week treatment period.

### 2.7. Outcome Assessment

Two blinded physiotherapists who had more than 3 years of relevant experience in neurological rehabilitation were responsible for conducting the outcome evaluations. Evaluations were performed at three time points: at baseline (week 0), postbrain stimulation (week 2; within 24 hours after the last rTMS/sham rTMS with task practice session), and follow-up (week 12; within 24 hours after the final task practice session).

A battery of assessment tools was chosen to evaluate the participants across the domains of International Classification of Function, Disability, and Health (ICF) (i.e., body functions/structures, activity, and participation) [35] (WHO 2001). The upperextremity portion of the FMA [36, 37] was the primary measure of arm motor impairment, whereas the MEP amplitude was the primary physiological outcome. Both of these were measures of body functions/structures. Secondary measures were grip force (measure of body functions/structures) [38–40], several measures of activity, including the Action Research Arm Test (ARAT) [41], nine-hole peg test (NHPT) [36, 40], box and block test (BBT) [40], and reaction time test (RT) [42], and a measure of participation (Stroke Impact Scale (SIS)) [43].

The peak-to-peak amplitude of the MEP of the FDI was measured as an indicator of motor cortex excitability [16]. Ten averaged MEPs evoked from the M1 hotspot on both the ipsilesional and contralesional sides were recorded, using a stimulation intensity of 120% of the rMT value measured at baseline. The same stimulator output was used for subsequent assessment sessions. For those participants whose MEP of the paretic hand could not be triggered at baseline despite the use of 100% stimulator output (MEP-status), a stimulator output of 100% was also used for subsequent outcome assessment sessions to measure the MEP amplitude. The FMA was used to assess the degree of motor impairment of the paretic upper limb (score range: 0-66) [36, 37].

Grip strength of the paretic hand was evaluated using the Jamar dynamometer (Sammon Preston Rolyan, Nottinghamshire, UK), according to the standard position and guidelines of the American Society of Hand Therapists [38–40]. The mean value of the force (in kg) recorded in three trials was used for analysis. The 19-item ARAT was used to assess various aspects of upper limb function (i.e., pinch, grip, grasp, and gross motor) (score range: 0-57 points) [36, 41]. The NHPT was used to evaluate finger dexterity (supplementary III) [36, 40]. If one was unable to perform the test within 10 minutes despite the best effort, a value of 600 s would be entered for data analysis purpose. The BBT measures the gross manual activity of the upper limb [40]. The number of blocks transferred over the partition from one side of the compartment to the other within a one-minute time period was recorded. The simple reaction time was also tested (supplementary III) [42]. The average of five trials was used for analysis. Finally, the 59-item SIS was used as a measure of self-reported health status (score range: 0-100) [43].

In each assessment and treatment session, the researcher also asked the participant whether he/she was receiving any concurrent therapies. Information regarding other therapies received during the intervention period was important because it may confound the effects of the experimental treatment.

### 2.8. Statistical Analysis

SPSS 21.0 for Windows (IBM, Armonk, NY, USA) was used for statistical analysis. Normality was checked using the Kolmogorov-Smirnov test. The demographic characteristics of the participants and outcome variables at baseline were compared using the Chi-square test, Mann-Whitney *U* test, and independent *t*-test as appropriate. For those participants with MEP-status at baseline who became MEP+ in posttest and follow-up assessments, a zero baseline value and the actual MEP amplitude value recorded in subsequent assessment sessions would be used for data analysis. Interhemispheric asymmetry (IHA) was calculated by the following formula [44]:

(1) $$\begin{array}{c} \text{IHA}=\frac{\left({\text{MEP}}_{\text{Paretic}}-\text{ME}{\text{P}}_{\text{Nonparetic}}\right)}{\left({\text{MEP}}_{\text{Paretic}}+\text{ME}{\text{P}}_{\text{Nonparetic}}\right)}. \end{array}$$

A lower MEP on the paretic side relative to the nonparetic side would generate a more negative IHA value. A less dramatic value (i.e., closer to 0) suggests a shift back to normalization (symmetry) [44].

For each continuous outcome measure (MEP, IHA, FMA, grip strength, ARAT, BBT, and reaction time test), a two-way ANOVA (mixed design; within-subject factor: time; between-subject factor: group) was used to determine whether there was a significant treatment effect (i.e., significant group × time interaction effect). Post hoc analysis was performed to examine the within-group changes using paired *t*-tests and between-group differences in change scores using independent *t*-tests.

For analysis of the NHPT, nonparametric statistics were used. The Wilcoxon test was used to examine the within-group changes over time, while the Mann-Whitney *U* test was used to examine the difference between the two groups at weeks 2 and 12.

Next, for those clinical outcome variables that yielded a significant treatment effect (i.e., significant group × time interaction effect), Pearson's product-moment correlation coefficients were used to determine the degree of correlation between changes in these variables and those in MEP amplitude on both sides and IHA.

Intention-to-treat analysis was first conducted. Any missing data would be substituted using the last-observation-carried-forward method. The alpha was set at 0.05, except for post hoc tests (alpha = 0.025 after Bonferroni correction) because of the two comparisons made (within-group analysis: baseline vs. week 2; week 2 vs. week 12).

## 3. Results

### 3.1. Participant Characteristics

The recruitment period was between 1 November 2015 and 30 November 2016. Twenty-four out of 187 patients screened fulfilled all eligibility criteria and were randomized to the experimental group (*n* = 12) or the sham control group (*n* = 12). One participant from each group withdrew during the course of the study. The remaining 11 participants in each group completed all assigned interventions and outcome assessments as scheduled (Figure 1). The participants did not report any adverse events during the study period. None of the participants received other concurrent therapies during the intervention period.

The two treatment arms were not significantly different in any demographic and clinical characteristics except for the MEP amplitude on both sides and BBT scores (Table 1). Among these three variables, the BBT was only a secondary outcome whereas the MEP amplitudes on both sides were considered as primary outcomes. Moreover, the between-group difference in MEP amplitude of the paretic hand yielded the smallest *p* value (*p* = 0.002), compared with BBT and MEP of the nonparetic hand. The MEP amplitude of the paretic hand was also correlated with the other two variables. Therefore, only the baseline MEP amplitude of the paretic hand was used as the covariate for subsequent analysis of covariance (ANCOVA) to assess the treatment effect on all the outcome variables. Although all not outcome variables were normally distributed, ANCOVA was used because it is robust against normality violations when the sample sizes are the same or very similar (i.e., both intervention and control groups: *n* = 12) [45].

### 3.2. Effect on Primary Outcomes

The intention-to-treat analysis detected a significant group × time interaction effect on MEP amplitude of the paretic hand (*F* = 5.116; *p* = 0.034), IHA (*F* = 4.396; *p* = 0.026), and FMA (*F* = 4.560; *p* = 0.045) (Table 2). Post hoc analysis showed that the increase in MEP amplitude of the paretic hand (*p* = 0.007), IHA (*p* = 0.012), and FMA (*p* = 0.004) was significantly different between the two groups during the 2-week stimulation period (Table 2).

The group × time interaction effect on MEP amplitude of the nonparetic hand demonstrated a trend (*F* = 4.298; *p* = 0.051) (Table 2). The between-group difference in change of this outcome variable during the whole 12-week period was significant (*p* = 0.005).

Further analysis was conducted to determine whether the change in the above outcomes obtained in the initial 2-week intervention period was well maintained after the 10-week follow-up period. The FMA scores continued to improve for both groups (*p* ≤ 0.025), but the changes were similar between them (*p* = 0.541). The changes in MEP amplitude of the paretic and nonparetic side during this period were not significant (*p* > 0.05) and demonstrated no significant between-group difference (*p* > 0.05), except a trend of a greater change (increase) of the MEP amplitude of the nonparetic hand in the control group (*p* = 0.090). The change of IHA during the follow-up period continued to show significant between-group difference (*p* = 0.012).

### 3.3. Effect on Secondary Outcomes

There were significant time × group interaction effects on ARAT (*F* = 7.809; *p* = 0.011) and BBT (*F* = 6.227; *p* = 0.021), but not grip strength (*F* = 0.020, *p* = 0.889), RT (*F* = 2.894; *p* = 0.104), or SIS (*F* = 0.078, *p* = 0.782) (Table 2). Post hoc tests showed that the changes in ARAT (*p* = 0.002) and BBT (*p* = 0.005) scores were significantly greater in the experimental group than the control group after the initial 2-week intervention period (Table 2).

The ARAT and BBT scores observed in the experimental group during the initial 2-week period showed no further changes during the subsequent follow-up period (*p* > 0.05). The changes in all the secondary outcome variables also demonstrated no significant between-group difference during the follow-up period (*p* > 0.05).

Regarding the NHPT performance, both groups showed significant improvement in NHPT performance following the intervention period (Table 2). No significant between-group differences in NHPT performance were noted at baseline (*p* = 0.734), the end of week 2 (*p* = 0.814), or week 12 (*p* = 0.487) (Table 2).

Overall, the per-protocol analysis revealed similar results, except that a significant group × time interaction effect was found with MEP of the nonparetic hand (*F* = 4.422; *p* = 0.049).

### 3.4. Association between Cortical Excitability and Functional Outcomes

Further analyses were conducted to examine whether MEP amplitude and IHA changes were correlated with the changes FMA, ARAT, and BBT scores, which were the three functional outcomes that showed a significant treatment effect (i.e., group × time interaction) in both the intention-to-treat and per-protocol analyses.

**Figure 3:**
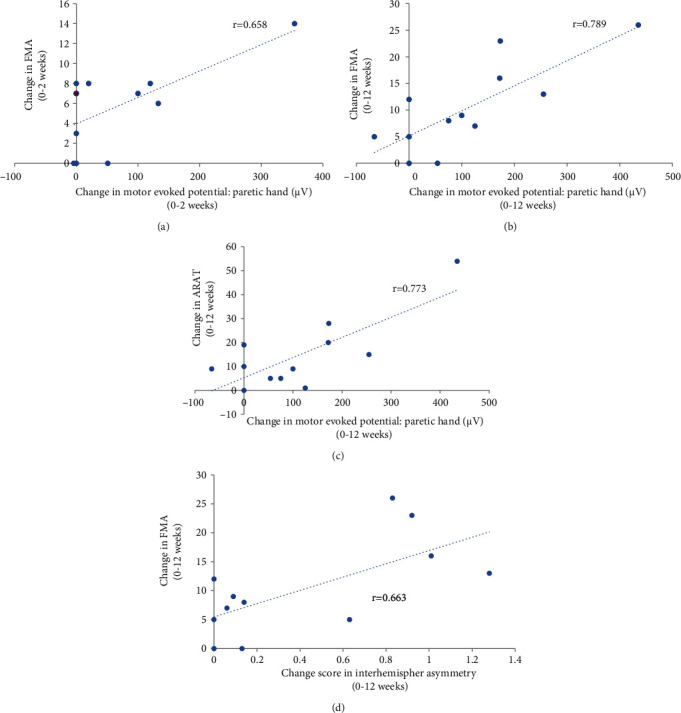
Association of changes in MEP amplitude and interhemispheric asymmetry with functional outcomes (*n* = 12). In the experimental group, the change in MEP amplitude over the 2-week stimulation period was significantly correlated with change in FMA (*r* = 0.658, *p* = 0.020) (a). Two participants who had the same combination of FMA and paretic hand MEP (a) values are indicated by the red dot. The change in MEP on the paretic side was correlated with that in FMA (*r* = 0.789, *p* = 0.002) (b) and ARAT scores (0.773, *p* = 0.003) (c) over the 12-week study period. The change of interhemispheric asymmetry was also correlated with that in FMA (*r* = 0.663, *p* = 0.019) over the 12-week study period (d).

In the experimental group, the increase in MEP amplitude of the paretic hand was significantly correlated with improvements in FMA score over both the 2-week stimulation (Figure 3(a)) and 12-week overall study periods (*p* < 0.05) (Figure 3(b)). The improvements in ARAT score during the 12-week study period were also correlated with the MEP amplitude changes over the same time period (*p* = 0.003) (Figure 3(c)). The increase of IHA in the experimental group was significantly correlated with the change of FMA over the 12-week period (*p* = 0.019) (Figure 3(d)). After removal of the participant who showed the greatest MEP changes, the correlation between FMA and MEP (paretic hand) (*r* = 0.639, *p* = 0.034) and IHA (*r* = 0.653, *p* = 0.029) changes over the 12-week study period remained significant (Supplementary file IV). Moreover, the association between IHA and ARAT changes was significant (*r* = 0.628, *p* = 0.039) (Supplementary file IV). These associations were not found in the sham control group. The changes in BBT scores were not significantly correlated with the MEP amplitude or IHA changes in both groups.

## 4. Discussion

### 4.1. Change in Outcomes with rTMS

The results indicated that inhibitory rTMS preceding repetitive motor task practice could effectively increase the excitability of the ipsilesional M1 (by an average of 24.9%) while suppressing the increase in excitability of the contralesional M1, resulting in a reduction in interhemispheric asymmetry of cortical excitability as reflected by the significant interaction effect on IHA values. Our experimental intervention also induced significantly better recovery of upper limb function than in the control group, as measured by the FMA, ARAT, and BBT at the end of the 2-week stimulation period. The average amount of improvement in FMA, ARAT, and BBT (5.7 points, 8.9 points, and 15 blocks, respectively) during this period (Table 2) exceeded their respective minimally clinically important difference values (5.2, 5.7 points, and 5.5 blocks, respectively) [37, 40, 41], indicating that the improvement attained was clinically important.

Our positive motor recovery findings are consistent with some previous studies on subacute stroke [13, 14, 19, 20]. Our results lend support to the notion that the inhibitory rTMS of the contralesional motor cortex may “prime” the affected motor cortex to boost use-dependent plasticity [16]. Previous work has shown that inhibitory rTMS induced reduction of the GABAergic-mediated interhemispheric inhibition from the contralesional to the ipsilesional hemisphere and an increase of excitability in the ipsilesional M1 [18]. After-effects in cortical excitability, probably through mechanisms that alter synaptic strength such as long-term potentiation, may last up to 2 hours [18, 46]. During this period when the inhibitory influence of the contralesional hemisphere on the ipsilesional hemisphere is reduced, the activity of the surviving neurons in ipsilesional M1 may be enhanced. There may also be unmasking of local and distant latent neural networks [4]. A more permissive environment is thus created for cortical reorganization to occur in response to the subsequent motor task practice that promotes use-dependent plasticity, which is essential in motor recovery poststroke.

Our results, however, are in discordance with two published subacute stroke studies by Tosun et al. [15] and Seniów et al., [21], which reported no between-group difference in functional outcomes following inhibitory rTMS combined with motor training. The discrepancies in findings may be explained by several reasons. First, recent evidence suggests that whether a true “imbalance” in interhemispheric inhibition exists in the human brain after stroke remains controversial [23]. Second, the interhemispheric inhibition model is an oversimplification and the effect of rTMS may vary depending on many factors [47].

Severity of stroke may be one of the factors. Inhibitory rTMS may be less effective for those with severe stroke [23, 47, 48]. The vicariation model holds that activity in the unaffected hemisphere is likely to play an important role in motor recovery, especially among those with larger stroke lesions on the ipsilesional side [23, 47]. According to the bimodal balance recovery model, the interhemispheric competition framework may be more relevant for driving recovery among those with less severe damage to the motor system of the lesioned hemisphere (i.e., more structural reserve) while the vicariation model may be a more viable option for supporting recovery among those with limited structural reserve in the lesioned hemisphere [49]. The participants in both Tosun et al. [15] and Seniów et al. [21] had considerably more severe motor impairment (mean FMA score = 26 and 38, respectively) than ours (mean = 48). Therefore, inhibiting the contralesional hemisphere through application of low-frequency rTMS in these severely impaired patients may not be an optimal method to promote recovery, according to the vicariation model. Indeed, Lin et al. identified a threshold of the clinical score (upper limb FMA = 43), above which, better motor performance in stroke patients is associated with lower transcallosal inhibition from the contralesional hemisphere, vice versa [50]. This may explain why positive results were obtained by applying inhibitory rTMS to the contralesional hemisphere among our participants with mild to moderate motor impairment.

The motor training program used may also influence the outcome. According to the framework proposed by Harris-Love and Harrington, task attributes are also an important determinant of treatment outcomes [49]. The type of task practiced should be matched to the targeted cortical site [49]. The tasks used in this study demand the acquisition and refinement of fine motor skills of the paretic hand, which should heavily involve M1 of the lesioned hemisphere, an area that has direct corticospinal projections to the distal muscles. On the other hand, Tosun et al. [15] used neuromuscular electrical stimulation of the wrist and finger extensors, rather than the active fine motor tasks used here. This may also explain their negative findings.

The positive findings in our study may also be partially attributable to the fact that our participants were in the subacute stage. The results of previous inhibitory rTMS trials in chronic stroke seemed to be more mixed than subacute stroke [16, 30, 51–53]. The stroke phase can determine the brain state and thus highly influence the plastic changes that are ongoing or already accomplished [47], which may render chronic patients less responsive to inhibitory rTMS relative to their counterparts in the subacute phase [53]. More research is required before a solid conclusion can be made regarding the relative effectiveness of inhibitory rTMS on cortical excitability and motor recovery in subacute versus chronic stroke patients.

The site of location (i.e., cortical or subcortical) could also influence the treatment effects of rTMS. A recent study by Kim et al. revealed that low-frequency rTMS applied to the contralesional hemisphere resulted in improvements in BBT and hand motor control only in the group without cortical involvement [54]. There are several potential explanations. First, structural or functional disconnection in cortical stroke could hinder the signal propagation among motor networks around the ipsilesional M1 [55, 56]. Second, the reduced effects of rTMS in cortical stroke could be attributed to greater suppression of GABA-ergic intracortical inhibition [57] and consecutive downregulation of GABA receptors in both hemispheres [58]. As the majority of our participants (91.3%) had subcortical stroke only, the treatment effect may be more apparent.

Not all the motor function outcomes measured here were enhanced by rTMS. The change in grip strength demonstrated no between-group difference. The focus of our task practice regimen was training of fine motor skills, rather than muscle strengthening. According to previous animal research, skill training induced plastic changes in cortical circuitry [59] whereas strength training did not effectively induce reorganization for movement representation in the cortex [17].

Besides grip strength, other secondary measures (NHPT, RT, and SIS) also did not demonstrate any between-group differences at different time points. A substantial number of individuals failed to perform the NHPT test. As a result, the median rather than the mean scores was used in the analysis, which necessitated the use of nonparametric tests, which are less powerful than parametric statistics [60]. Only the primary outcomes (MEP and FMA) were used to estimate the sample size. This study may be underpowered to assess the treatment effect on some of the secondary outcomes.

### 4.2. Maintenance of Treatment Effect during Follow-Up Period

Our results firstly indicated that the improvement in the MEP (paretic hand), IHA, ARAT, and BBT after the 2-week stimulation period was well maintained with continued motor task practice, despite the termination of the rTMS. To our knowledge, this is the first study to show that the effects of rTMS combined with motor task practice on MEP of paretic side and IHA are sustained for more than 2 months. Three previous studies incorporated a follow-up period of more than 2 months following inhibitory rTMS in subacute stroke patients. Blesneag et al. found that following 10 days of low-frequency rTMS intervention starting at 10 days poststroke onset, there was a trend in decreased excitability of the contralesional hemisphere and a trend in increased excitability of the lesioned hemisphere measured at 45 days poststroke onset. A tendency toward balanced interhemispheric excitability was also detected at 90 days poststroke onset. Their findings thus indicated that low-frequency rTMS has a sustained treatment effect on cortical excitability on both sides. Overall, their findings on cortical excitability are largely similar to ours. However, their study did not include any motor task practice immediately after rTMS, nor during the follow-up period [26]. Lüdemann-Podubecka et al. found a significant long-term effect on MEP of the nonparetic side at 6-month follow-up. However, the MEP of the paretic side was not measured, and thus IHA could not be evaluated [25]. In another study involving patients with more severe stroke, Seniów et al. did not find any immediate or long-term effects on motor outcomes, nor was MEP measured [21].

The similar and significant increase in FMA scores observed in both groups during the follow-up period can be attributable to repetitive motor task practice in our study. It is not known whether the improvement gained in the initial stimulation period can be maintained without the continued task practice. It cannot be ruled out that the motor task practice during the follow-up period could have potentially washed out the specific effects of rTMS-primed training. However, this is quite unlikely because the data from the control group showed that the motor practice with sham rTMS (week 0-2) or the motor practice alone (week 2-12) had relatively little effects.

### 4.3. Association between Cortical Excitability and Upper Limb Function

Another new finding of this study is that the FMA/ARAT scores were associated with an increase in MEP magnitude on the paretic side and reduced IHA over the 12-week study period. Du et al. [13] also reported an association between improvement of FMA score and rMT change in the ipsilesional hemisphere following five treatment sessions in a group of acute and subacute stroke patients. However, their correlation (*r* = 0.615) was based on the combined data of participants who had received low-frequency rTMS over the contralesional hemisphere, and those who had received high-frequency rTMS over the ipsilesional hemisphere. Our results have to be interpreted with caution because one of the participants had considerably greater changes in MEP amplitude and upper limb motor function than others. However, despite removing this participant from the analysis, certain correlations remained significant (e.g., FMA with paretic hand MEP and IHA changes over the 12-week study period) (Supplementary IV). Regarding the relationship between treatment efficacy on IHA and functional recovery in subacute stroke, previous research is scarce. In a cross-sectional correctional study, Brouwer and Schryburt-Brown found significant relationship between IHA and simple hand movement (e.g., tapping) in subacute stroke patients [44]. Later, Wang et al. [61] found that two weeks of low-frequency rTMS improved the IHA and lower limb function, but they did not investigate the correlation between them. The results of this study are thus novel in that it provides some evidence of a positive association between upper limb functional improvement and IHA following low-frequency rTMS combined with a structured motor task practice program. Our results thus lend support to the notion that improvement in motor function through task-specific training was associated with neuroplastic changes [62, 63] and that the location of cortical reorganization corresponded to the specific areas being used [64].

### 4.4. Limitations

The findings of this study can only be generalized to patients who have similar demographic and clinical characteristics as our study participants (i.e., subacute stroke patients aged ≥60 years with mild to moderate arm paresis). This trial was a single-center study, and the sample size was small. This may explain the nonsignificant findings in some of the outcomes. Also, subgroup analysis could not be conducted to explore the influence of stroke lesion location on outcomes. A multicentered study with a larger sample size may be required to further confirm the results and decipher the effects of different patient characteristics on treatment effect.

Despite random allocation of groups, the MEP on both sides demonstrated significant between-group differences at baseline, which may confound the results. However, we used the baseline paretic hand MEP amplitude as a covariate in all the ANCOVA models to account for the baseline differences. Holding a coil on the edge produces less tactile sensation than when it is tangential to the scalp. It is thus possible that participants could detect differences that might lead them to have different expectations of effect. Considering that all participants had never experienced rTMS before, it is unlikely that this would have unblinded rTMS intervention [13]. However, the sham stimulation method used here remains a limiting factor of the study.

We used the MEP values on each side to calculate the degree of asymmetry of cortical excitability. However, we did not measure the interhemispheric inhibition directly. It is possible that the asymmetry observed stems from mechanisms other than imbalance in interhemispheric inhibition.

The possibility that the motor task practice during the follow-up period could have potentially washed out the specific effects of rTMS-primed training could not be ruled out. Finally, the task practice program was standardized across the participants throughout the study period. While it minimized the confounding effect of different motor practice parameters (e.g., intensity and duration) on the results, the lack of personalized training and progression pattern may not be optimal for achieving the best possible outcomes for individual participants with different degree of motor deficits.

## 5. Conclusion

This study showed that 10 sessions of low-frequency rTMS preceding repetitive upper limb motor task practice was effective in increasing the excitability of the ipsilesional motor cortex, reducing the interhemispheric asymmetry, and improving motor function among subacute stroke patients aged ≥60 years with mild to moderate arm paresis. The improvement gained was well maintained after another 10 weeks with continued task practice without rTMS. The gain in motor function was associated with an increase in cortical excitability of the ipsilesional motor cortex and reduction in IHA. Thus, the results seem to support the interhemispheric competition theory in explaining motor recovery of people with less severe subcortical stroke.

## Figures and Tables

**Table 1:** Demographic and clinical characteristics of participants.

Variable	Experimental (*n* = 12)	Control (*n* = 12)	*p* value
*Demographic and clinical characteristics*			
Age (years)	67.3 (5.8)	65.1 (3.1)	0.216
Time since stroke (weeks)	13.6 (6.1)	15.1 (7.0)	0.582
Gender (female : male) (*n*)	5 : 7	5 : 7	1.000
Paretic limb (right : left) (*n*)	4 : 8	6 : 6	0.408
Type of stroke (infarct : hemorrhage) (*n*)	12 : 0	11 : 1	0.307
Paretic hand (dominant : nondominant) (*n*)	4 : 8	6 : 6	0.408
Site of lesion (subcortical only : cortical involvement, *n*) (*n*)	12 : 0	9 : 3	0.217
Resting motor threshold (paretic hand) (% stimulator output)	85.5 (14.5)	80.6 (12.7)	0.320
Resting motor threshold (nonparetic hand) (% stimulator output)	77.3 (8.3)	72.6 (12.3)	0.524
*Primary outcome measures*			
Fugl-Meyer motor assessment	46.7 (13.9)	48.8 (14.9)	0.549
MEP amplitude (paretic hand) (*μ*V)	258.3 (278.0)	600.1 (374.6)	0.002^∗^
MEP amplitude (nonparetic hand) (*μ*V)	316.8 (229.4)	693.5 (380.3)	0.035^∗^
IHA	-0.39 (0.67)	0.06 (0.39)	0.155
*Secondary outcome measures*			
Grip strength (kg)	12.6 (11.3)	13.8 (10.8)	0.792
Action Research Arm Test	36.5 (17.8)	41.1 (17.8)	0.332
Nine-hole peg test (median (IQR)) (s)	351 (553)	328 (565)	0.734
Box and block test (number of blocks)	17.2 (20.5)	33.8 (18.7)	0.049^∗^
Reaction time (ms)	985.8 (439.9)	654.2 (285.5)	0.068
Stroke Impact Scale	59.0 (13.3)	65.7 (10.3)	0.141

Note: mean (SD) presented unless indicated otherwise; MEP: motor-evoked potential; IHA: interhemispheric asymmetry; IQR: interquartile range. ^∗^Significant between-group difference (*p* ≤ 0.05).

**Table 2:** Outcome measures (*n* = 24).

Outcome	Experimental group (*n* = 12)						Control group (*n* = 12)					
	Baseline	Week 2	Week 12	Change (week 2–baseline)	Change (week 12–week 2)	Change (week 12–baseline)	Baseline	Week 2	Week 12	Change (week 2–baseline)	Change (week 12–week 2)	Change (week 12–baseline)
FMA^∗^	46.7 (13.9)	52.3 (11.9)^†^	57.0 (9.6)^‡^	5.7 (4.3)^#^	4.7 (6.3)	10.2 (8.2)	48.8 (14.9)	49.9 (15.3)	53.3 (16.4)^‡^	1.2 (2.3)	3.3 (4.0)	4.5 (4.5)
MEP (paretic hand) (*μ*V)^∗^	258.8 (278.0)	323.3 (279.5)	369.1 (244.6)	64.5 (104.7)^#^	45.8 (78.5)	110.3 (136.9)^#^	600.1 (374.6)	565 (371.7)	586 (384.9)	-35.1 (51.8)	21.9 (70.9)	-13.2 (72.3)
MEP (nonparetic hand) (*μ*V)	316.8 (229.4)	301.8 (179.5)	258.4 (197.1)	-15.1 (219.1)	-43.3 (96.7)	-58.4 (264.6)^#^	693.5 (380.3)	886.4 (86.5)	1072.9 (72.9)	192.9 (326.5)	186.5 (422.0)	379.4 (414.7)
IHA^∗^	-0.39 (0.67)	-0.24 (0.62)	0.04 (0.55)^‡^	0.15 (0.27)^#^	0.27 (0.38)^#^	0.42 (0.47)^#^	-0.06 (0.39)	-0.15 (0.41)	-0.21 (0.41)^‡^	-0.09 (0.12)	-0.07 (0.15)	-0.15 (0.14)
Grip strength (kg)	12.6 (11.3)	13.9 (10.8)	15.4 (10.1)^‡^	1.3 (2.7)	1.5 (1.9)	2.8 (3.5)	13.8 (10.8)	13.9 (10.5)	14.4 (11.1)	0.1 (2.6)	0.5 (2.1)	0.6 (1.1)
ARAT^∗^	36.5 (17.8)	45.4 (11.4)^†^	51.1 (8.9)	8.9 (7.5)^#^	5.7 (9.5)	14.6 (14.9)^#^	41.1 (17.8)	41.1 (17.9)	43.2 (17.9)	0 (1.7)	2.1 (3.9)	2.1 (4.2)
NHPT: median (IQR)	351 (553)	71 (558)^§^	51 (85)	—	—	—	328 (565)	165 (573)^§^	142 (485)	—	—	—
Box and block test^∗^	17.2 (20.4)	32.3 (17.7)^†^	34.8 (14.8)	15.2 (14.6)^#^	2.5 (9.4)	17.7 (12.9)	33.8 (18.7)	34 (19.7)	41.8 (20.9)^‡^	0.2 (7.0)	7.8 (9.1)	7.9 (8.0)
Reaction time (ms)	985.8 (439.9)	902.2 (398.6)	837.3 (309.1)	-83.7 (131.5)	-64.8 (222.3)	-148.5 (301.1)	654.1 (285.5)	651.7 (285.4)	637.9 (291.2)	-2.5 (50.5)	-13.8 (132.0)	-16.3 (86.9)
Stroke Impact Scale	59.0 (13.3)	64.1 (8.5)	68.4 (6.9)^‡^	5.0 (7.5)	4.7 (6.3)	9.4 (9.1)	65.9 (10.3)	69.6 (11.2)^†^	72.7 (11.6)^‡^	3.6 (3.9)	3.1 (3.2)	6.7 (5.7)

Mean (SD) presented unless indicated otherwise; FMA: Fugl-Meyer assessment; MEP: motor-evoked potential; *μ*V: microvolt; IHA: interhemispheric asymmetry; ARAT: Action Research Arm Test; NHPT: nine-hole peg test; IQR: interquartile range; ^∗^significant group × interaction effect for the 2-week intervention period (*p* ≤ 0.05); ^†^significant difference between baseline and week 2 (paired *t*-test, *p* ≤ 0.025); IHA = (Paretic − Nonparetic)/(Paretic + Nonparetic); ^‡^significant difference between week 2 and week 12 (paired *t*-test, *p* ≤ 0.025); ^#^significantly different from the control group (independent *t*-test, *p* ≤ 0.025); ^§^significant difference between baseline and week 2 (Wilcoxon test, *p* ≤ 0.025).

## Data Availability

The data that support the findings of this study are available from the corresponding author upon reasonable request.

## Supplementary Materials

Supplementary 1 Supplementary I: Sample size calculation.

Supplementary 2 Supplementary II: Figure 1: neuronavigation system and identification of hotspot.

Supplementary 3 Supplementary III: Nine-hole peg test (NHPT) and testing of reaction time.

Supplementary 4 Supplementary IV: Association of changes in MEP amplitude, interhemispheric asymmetry with functional outcomes (*n* = 11).
