# Supplementary material for: Low-Frequency rTMS over Contralesional M1 Increases Ipsilesional Cortical Excitability and Motor Function with Decreased Interhemispheric Asymmetry in Subacute Stroke: A Randomized Controlled Study
Source: Neural Plast. 2022 Jan 5;2022:3815357. doi: 10.1155/2022/3815357 (PMC8756161; doi:10.1155/2022/3815357)
Supplement: Supplementary 1 — Supplementary I: Sample size calculation. [file 3815357.f1.docx]

**Supplementary Ⅰ： Sample size calculation**

The sample size was estimated from published data on motor evoked potential (MEP) (i.e., primary physiological outcome), and Fugl-Meyer Motor Assessment (FMA) scores (i.e., primary behavioral outcome). In a study involving a sample of 20 people by Takeuchi^1^, a significant improvement in MEP was found in the intervention group, with an effect size of d=0.6 (equivalent to f=0.30). In Zheng et al.^2^, the rTMS treatment yielded a mean FMA change of 13.2, SD=7.2), translating to an effect size of Cohen’s d=1.82 (equivalent to f=0.91). We used a more conservative approach by adopting f=0.30 as the assumed effect size. With a power of 80%, alpha of 0.05, and a 10% attrition rate, the minimum sample size of 24 individuals with stroke would be required to detect a significant group × time interaction effect using 2-way analysis of variance (ANOVA) with mixed design.

References:

1. Takeuchi N, Chuma T, Matsuo Y, Watanabe I, Ikoma K. Repetitive transcranial magnetic stimulation of contralesional primary motor cortex improves hand function after stroke. *Stroke*. 2005;36:2681-2686.
2. Zheng CJ, Liao WJ, Xia WG. Effect of combined low-frequency repetitive transcranial magnetic stimulation and virtual reality training on upper limb function in subacute stroke: a double-blind randomized controlled trail. *Curr Med Sci*. 2015;35:248-254.
