# Supplementary material for: Low-Frequency rTMS over Contralesional M1 Increases Ipsilesional Cortical Excitability and Motor Function with Decreased Interhemispheric Asymmetry in Subacute Stroke: A Randomized Controlled Study
Source: Neural Plast. 2022 Jan 5;2022:3815357. doi: 10.1155/2022/3815357 (PMC8756161; doi:10.1155/2022/3815357)
Supplement: Supplementary 2 — Supplementary II: Figure 1: neuronavigation system and identification of hotspot. [file 3815357.f2.pdf]

Supplementary II Figure 1_Neuronavigation system and identification of hotspot

A)

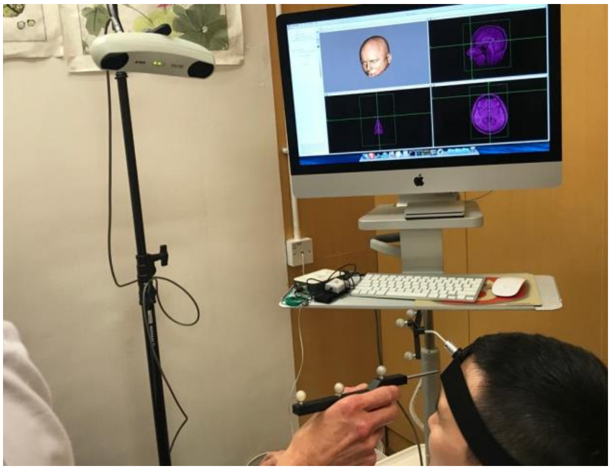

B)

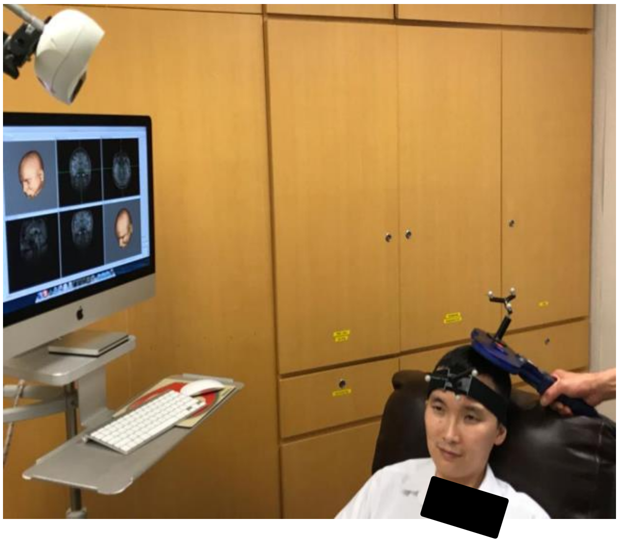

A) Skull model was recreated.
B) The hotspot was identified and marked using the neuronavigation system.
