# Supplementary material for: Low-Frequency rTMS over Contralesional M1 Increases Ipsilesional Cortical Excitability and Motor Function with Decreased Interhemispheric Asymmetry in Subacute Stroke: A Randomized Controlled Study
Source: Neural Plast. 2022 Jan 5;2022:3815357. doi: 10.1155/2022/3815357 (PMC8756161; doi:10.1155/2022/3815357)
Supplement: Supplementary 3 — Supplementary III: Nine-hole peg test (NHPT) and testing of reaction time. [file 3815357.f3.docx]

**Supplementary Ⅲ. Nine-Hole Peg Test (NHPT) and testing of reaction time**

**Nine-Hole Peg Test (NHPT):** A nine-hole pegboard was positioned at the midline in front of the participants. The participants were asked to remove the pegs from the container, one at a time, and place them into the holes on the board and take each peg back to the container, as fast as able. The time taken to complete the test (in seconds) was recorded using a stopwatch.


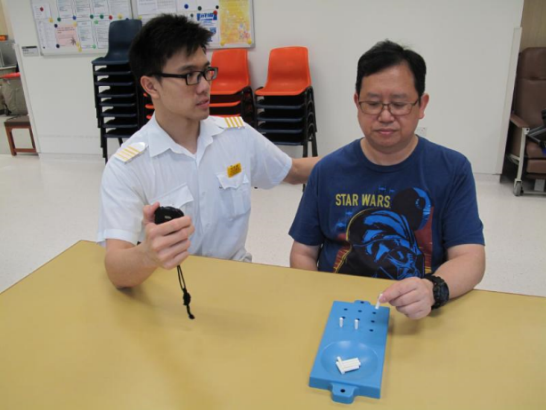


**Reaction time:** After a random time period (ranging from 180 ms to 200 ms) following the appearance of the image of an apple on the computer screen, the image of a dragon fruit would appear. Participants were required to press the space bar of the computer using the affected hand upon seeing the image of the dragon fruit on the screen. The time gap between the appearance of the dragon fruit and the participant pressing the space bar was recorded by the system.
