# Supplementary material for: Low-Frequency rTMS over Contralesional M1 Increases Ipsilesional Cortical Excitability and Motor Function with Decreased Interhemispheric Asymmetry in Subacute Stroke: A Randomized Controlled Study
Source: Neural Plast. 2022 Jan 5;2022:3815357. doi: 10.1155/2022/3815357 (PMC8756161; doi:10.1155/2022/3815357)
Supplement: Supplementary 4 — Supplementary IV: Association of changes in MEP amplitude, interhemispheric asymmetry with functional outcomes (n = 11). [file 3815357.f4.pdf]

Supplementary IV : Association of Changes in MEP amplitude and interhemispheric asymmetry with functional outcomes (n=11)

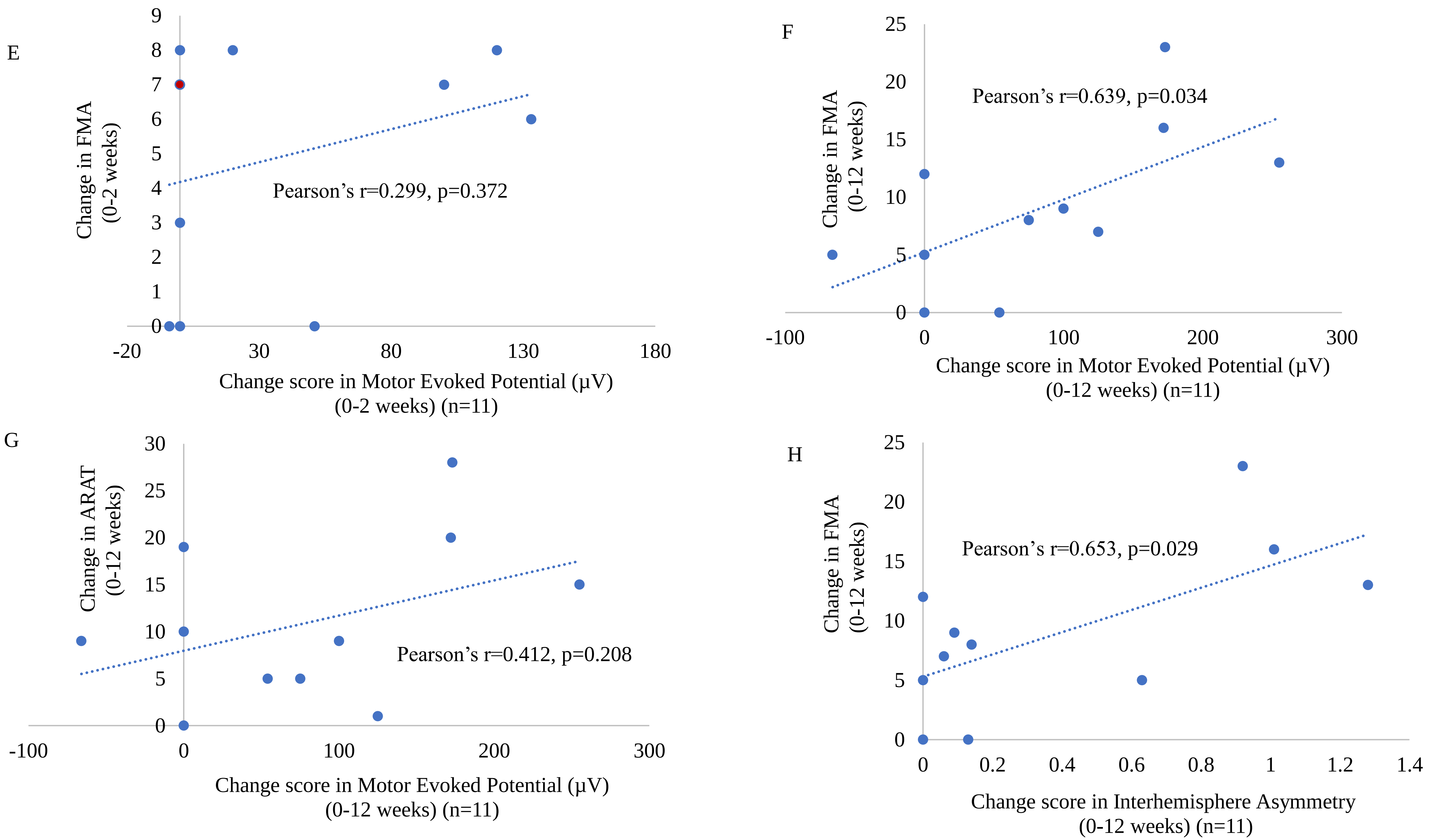

I

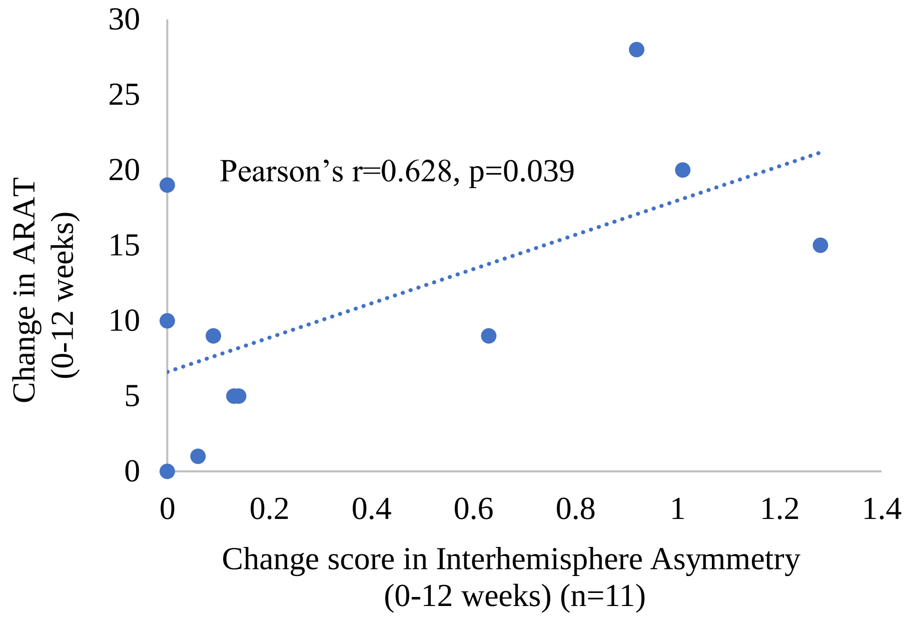

Correlation analysis was conducted after removal of one experimental group participant who showed the greatest MEP changes. Based on the data collected from 11 participants in the experimental group, the change in MEP amplitude over the 2-week stimulation period was no longer significantly correlated with change in FMA (r=0.299, p= 0.372) (E). Two participants who had the same combination of FMA and paretic hand MEP values are indicated by the red dot. The change in MEP on the paretic side remained significantly associated with that in FMA (r=0.639, p=0.034) (F) but not with ARAT scores (r=0.412, p=0.208) (G) over the 12-weeks study period. The change of interhemispheric asymmetry was still significantly correlated with that in FMA (r=0.653, p=0.029) (H) and ARAT scores (0.628, p=0.039) (I) over the 12-week study period.
